# Assessing Consciousness through Neurofeedback and Neuromodulation: Possibilities and Challenges

**DOI:** 10.3390/life13081675

**Published:** 2023-08-02

**Authors:** Martina Vatrano, Idan Efim Nemirovsky, Paolo Tonin, Francesco Riganello

**Affiliations:** 1 S. Anna Institute, Research in Advanced Neurorehabilitation, Via Siris, 11, 88900 Crotone, Italy; patonin18@gmail.com; 2 Department of Physics and Astronomy, Brain and Mind Institute, University of Western Ontario, London, ON N6A 3K7, Canada; idannemirov@gmail.com

**Keywords:** neurofeedback, disorders of consciousness, TMS, BCI, tDCS

## Abstract

Neurofeedback is a non-invasive therapeutic approach that has gained traction in recent years, showing promising results for various neurological and psychiatric conditions. It involves real-time monitoring of brain activity, allowing individuals to gain control over their own brainwaves and improve cognitive performance or alleviate symptoms. The use of electroencephalography (EEG), such as brain–computer interface (BCI), transcranial direct current stimulation (tDCS), and transcranial magnetic stimulation (TMS), has been instrumental in developing neurofeedback techniques. However, the application of these tools in patients with disorders of consciousness (DoC) presents unique challenges. In this narrative review, we explore the use of neurofeedback in treating patients with DoC. More specifically, we discuss the advantages and challenges of using tools such as EEG neurofeedback, tDCS, TMS, and BCI for these conditions. Ultimately, we hope to provide the neuroscientific community with a comprehensive overview of neurofeedback and emphasize its potential therapeutic applications in severe cases of impaired consciousness levels.

## 1. Introduction

Acquired brain injury (ABI) is a term that includes traumatic and non-traumatic causes of brain damage. It does not include brain injuries that are hereditary, congenital, or degenerative [1]. Common traumatic causes of ABI include car accidents, gunshot wounds, and sports injuries, while non-traumatic events include focal brain lesions, anoxia, tumors, aneurysm, vascular malformations, and infections [2,3]. Following the coma phase, these patients may be diagnosed with a Disorder of Consciousness (DoC). The most common diagnoses of DoC include Unresponsive Wakefulness Syndrome/Vegetative State (UWS/VS) and the Minimally Conscious State (MCS). UWS/VS is characterized by spontaneous eye opening, preserved autonomic function, and in some cases, reflexive behaviors [4]. On the other hand, patients in MCS present minimal but clear behavioral evidence of awareness of self or the environment, simple execution of commands, and intelligible responses that can be distinguished as “yes–no” (verbal or gestural). MCS patients that are able to follow commands are further diagnosed as MCS+, while patients who do not demonstrate command following are indicated as MCS− [5]. Emergence from MCS is characterized by the re-emergence of a functional communication system or restoration of the ability to use objects functionally. Recovery of communication is evidenced by reliable yes–no responses to questions about personal or situational orientation [6]. The current gold standard for diagnosing DoC involves repeated clinical evaluations using standardized scales such as the Coma Recovery Scale-Revised (CRS-R) [7]. These scales rely on observing several categories related to a patient’s spontaneous and stimulus-induced behaviors, and scores are assigned depending on their capacity to meaningfully respond to stimuli [8].

## 2. DoC and NeuroRehabilitation

Neurorehabilitation is a vast topic that includes the initial rehabilitation of patients with compromised conscious capacity to more advanced stages of recovery, such as the reintegration into social and occupational settings [9]. Neurological recovery after an ABI results from the brain’s plasticity and ability to repair and re-organize itself [10]. It can be promoted by rehabilitation programs (i.e., occupational and cognitive therapy), which are essential to cope with complex brain dysfunction and facilitate patient recovery. To activate neuroplasticity, the process of recovery should involve a task-specific approach that is repetitive, challenging, and motivating.

There is strong evidence to support intensive rehabilitation programs in cases of severe traumatic brain injury (TBI), which are linked to quicker functional improvements [11]. Some evidence suggests that initiating intervention during emergency and acute care could be beneficial. Group-based rehabilitation in a therapeutic environment has proven effective for patients needing neuropsychological rehabilitation after a severe brain injury [11]. Despite these findings, there are not any global standards for early rehabilitation treatment cases of severe TBI [11]. Moreover, randomized controlled trials fail to determine the most effective long-term treatments for individual patients or the most economical models for institutional long-term care [12]. Another review examined the evidence supporting the effectiveness of multidisciplinary rehabilitation following acquired brain injury in working-age adults. The authors concluded that TBI rehabilitation is a complex process that requires further research and development [13]. One particular challenge they reported is the difficulty of predicting recovery from disorders of consciousness due to individual differences in neuroplasticity. Beyond neurological differences, there are several health-related factors that impact the condition of patients and their prospects for recovery. These include other aspects of physical health (i.e., musculoskeletal injuries), as well as patients’ psychological and emotional states. Therefore, neurological rehabilitation is further complicated by the diversity of patients and their conditions.

The intensity and duration of therapy are two essential factors in devising treatment programs. Zhu and colleagues have found that TBI patients undergoing more intensive therapy tend to show quicker improvements [14]. However, a study by Hart and colleagues showed no significant improvement in functional or emotional outcomes when employing a longer and more intensive rehabilitation program [15].

Another approach is to provide more intensive therapy that could potentially shorten the length of stay (LOS) in rehabilitation facilities. For instance, Slade et al. demonstrated a 14-day reduction in LOS for a group undergoing more intensive treatment [16]. On the other hand, Formisano and colleagues reported that LOS in rehabilitation was strongly associated with significant improvements [17], which contradicts the approach based on intensive therapy. Furthermore, it was reported that longer rehabilitative treatments may have the benefit of reducing dependency and long-term care costs in some patients [18]. In either case, it is important to consider that LOS is influenced by various factors that relate to both individual patients and specific healthcare systems [18].

In the context of neurorehabilitation and continuing therapeutic developments, neurofeedback has emerged as a promising approach for DoC patients diagnosed as either UWS/VS or MCS [19,20,21]. Neurofeedback techniques leverage the brain’s inherent plasticity, aiming to restore or enhance neural function through targeted training and stimulation [22]. This is achieved by coupling the real-time monitoring of brain activity, typically via electroencephalography (EEG), with the feedback that promotes beneficial neural patterns. Neurorehabilitation encompasses a broad range of therapies designed to improve neurological function and patient quality of life. While these approaches offer promising benefits to diagnosis and recovery, they also present challenges to clinical implementations. The following sections present different neurofeedback techniques and outline in detail their potential uses, advantages, and limitations.

## 3. EEG Neurofeedback

The application of EEG in neurorehabilitation provides a non-invasive method for monitoring brain activity in real-time. This observation of cortical activity provides valuable insight into neural recovery processes, which can assist in the customization of therapeutic interventions for patients recovering from neurological injuries such as ABI. In the evaluation of patients with DoC, EEG has been an invaluable tool that can be used to assess residual cognitive abilities such as basic auditory capabilities [23] and hidden comprehension of speech [24], which may also aid in predicting patient outcomes.

EEG neurofeedback (NF) couples the observation of brain activity with feedback that can reinforce neural circuits important to cognitive and motor function [22]. By providing feedback on specific brainwave or connectivity patterns, individuals can learn to modulate their brain activity in a targeted manner that promotes plasticity and functional reorganization in the brain. This also involves the ability to modulate and control subconscious neural activity [22]. While EEG is a powerful technique, its use in DoC patients could be complicated by the presence of motion or sweating artifacts, making it difficult to obtain a clear signal.

**Figure 1:**
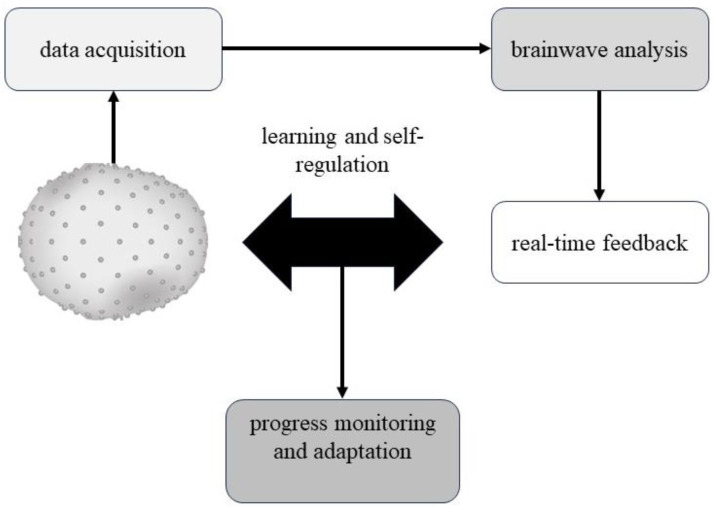
EEG neurofeedback. Representation of the five EEG neurofeedback: (1) data acquisition; (2) brain-wave analysis; (3) real-time feedback; (4) learning and self-regulation; and (5) progress monitoring and adaptation.

The NF process can be divided into several steps (Figure 1): (1) data acquisition; (2) brainwave analysis; (3) real-time feedback; (4) learning and self-regulation; and (5) progress monitoring and adaptation. In the first step, the data are acquired in real-time from the subject, placing electrodes on the scalp to measure the brain’s electrical activity. The signals are then converted into brainwave patterns that can be analyzed and interpreted. In the second step, the recorded EEG data are analyzed in real-time to identify specific brainwave patterns typically associated with various cognitive or emotional processes, such as attention, relaxation, or emotional regulation. In the third phase, once the relevant brainwave patterns have been identified, the subjects receive real-time feedback on their brain activity through visual or auditory cues. The fourth step consists of learning and self-regulation in which the subject, through repeated exposure to real-time feedback, learns to recognize and control their brain activity to achieve a desired outcome, such as improved attention, relaxation, or emotional regulation. These processes rely on the principles of operant conditioning and reinforcement, in which individuals are rewarded for the successful modulation of their brain activity. The last step consists of progress monitoring and adaptation. Throughout the programmed sessions, this step involves tracking progress and adapting the training protocol according to the patient’s needs.

**Figure 2:**
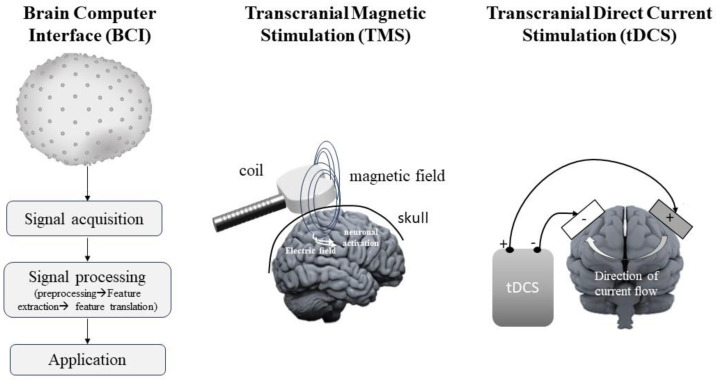
Scheme of BCI, TMS, and tDCS. A BCI based on EEG is a technique that allows for brain activity to be directly translated into motor actions and can provide patients with a means of communication and control. TMS is a non-invasive technique for brain stimulation. In TMS, a high-intensity electric current, which changes rapidly (TMS pulse), is directed through conducting wire loops enclosed in a protective casing and positioned against a specific area on the scalp. This alteration in the electrical current generates a strong, fluctuating magnetic field that can easily pass through the skull and stimulate a secondary electric current within the brain’s excitable tissues. tDCS is a non-invasive approach that delivers a weak direct current (usually 1–2 mA) to specific cortical regions beneath two opposing electrodes contained in saline-soaked sponges.

More generally, an important advantage of EEG is the ability to employ it alongside other techniques, such as a brain–computer interface (BCI) and transcranial magnetic stimulation (TMS), which we discuss in the following two sections of the review (Figure 2). While these are two common approaches to neurofeedback, another important method we discuss is transcranial direct current stimulation (tDCS), to which we dedicate the final section of this review (Figure 2).

### 3.1. BCI

A BCI based on EEG is a technique that allows for brain activity to be directly translated into motor actions and can provide a means of communication and control for patients with DoC who are unable to move or speak. BCI technology allows for direct communication between the brain and a computer or other devices without the need for physical movement or speech [25]. BCI works by detecting and translating the electrical signals generated by the brain into commands that allow patients to communicate yes/no answers to questions, select items on a screen, or even control devices such as wheelchairs or robotic arms [25]. Observing and eventually modulating specific patterns of brain activity through self-regulation is essential for developing and implementing BCIs for therapeutic purposes. Long-term neuroplasticity can be induced by training the brain to perform specific tasks, promoting the overall improvement of brain functions [26]. Several studies highlighted the potential of using BCI in DoC patients. Coyle et al. reported that by EEG evaluation, patients with minimal consciousness could potentially use the BCI communication system, even if they cannot voluntarily control their movements. The recognition of consciousness in this study was derived from unique sensorimotor patterns for each imagined motor task [27]. In another work, Li and colleagues [28] investigate the application of a combined BCI for identifying number processing and mental arithmetic in patients’ DoC. The combined BCI integrates multiple types of brain signals or different BCI paradigms to improve the performance, reliability, and robustness of the interface [29]. In this case, the BCI system was combined with P300 and steady-state visual evoked potentials (SSVEPs) to guide three MCS, six UWS/VS, and two patients who emerged from MCS patients through three tasks: recognizing numbers; comparing numbers; and performing mental arithmetic (addition and subtraction). Interestingly, two of the six patients diagnosed as UWS/VS performed with accuracies significantly greater than the chance level, emphasizing the potential to use the combined BCI system in identifying and evaluating covert cognitive abilities in the DoC [28].

In another study involving DoC patients, Xiao and colleagues applied BCI in the evaluation of the visual fixation [30]. Despite not demonstrating visual fixation as evaluated by the CRS-R, 1 of the 15 patients in this study demonstrated a significant online accuracy in the BCI assessment. Another promising approach to assess DoC patients was used by Pan and Colleagues [31] to identify cases of cognitive motor dissociation (CMD) with BCI. CMD is a condition where patients show no observable responses to commands despite demonstrating an appropriate response through neuroimaging. This study assessed 45 UWS/VS and 33 MCS patients who were asked to execute a cognitive task (such as recognizing and selecting a photograph). CMD was observed in 18 UWS/VS and 16 MCS patients, indicating the strong diagnostic potential of BCI as opposed to traditional scales that rely solely on behavior.

Another work by Xie and colleagues [32] employed a gaze-independent audiovisual BCI system, which was used to assess the capacity of eight DoC patients to pay attention to congruent and incongruent visual stimuli presented sequentially. These stimuli typically evoke event-related potentials (ERPs), which were used as indicators of capability in this study. Notably, three patients demonstrated the ability to follow commands and recognize numbers, emphasizing the ability of this system to detect awareness levels in patients with DoC.

The possibility for DoC patients to follow commands was also studied by Lulé et al. [33], who used EEG-based BCI in a group of 18 DoC patients. Command-following was detected in one patient with MCS and another patient with locked-in syndrome (LIS).

While the performance of BCIs in assessing the consciousness level of DoC patients appears promising, their effectiveness varies widely depending on the type of BCI and paradigm used. Several studies used P300-based BCIs [34,35,36], which were shown to be effective when assessing consciousness in DoC patients, even when no behavioral responses were observed. Other studies investigated the use of vibrotactile BCIs to detect consciousness [37,38,39,40], which showed promising results as some patients demonstrated neurophysiological signs of command following in the absence of behavioral responses. The results with Hybrid BCIs, which combine multiple EEG markers such as P300 and SSVEPs [31,41], indicated the potential benefits of a multimodal approach to the assessment of consciousness in DoC patients. On the other hand, BCIs based on sensorimotor rhythms showed limited success in assessing consciousness in these patients, highlighting the need for more research in this domain [27,42]. The results of BCI studies are summarized in Table 1.

### 3.2. TMS

TMS, a non-invasive technique for brain stimulation, was initially introduced by Barker and colleagues in 1985 [43]. It is considered safe when used in accordance with the safety and application guidelines approved by the International Federation of Clinical Neurophysiology [44]. This technique is based on Faraday’s principle of electromagnetic induction [45].

In TMS, a high-intensity electric current, which changes rapidly (known as the TMS pulse), is directed through conducting wire loops enclosed in a protective casing and positioned against a specific area on the scalp. This alteration in the electrical current generates a strong, fluctuating magnetic field that can easily pass through the skull and stimulate a secondary electric current (moving in the opposite direction to the initial current) within the brain’s excitable tissues [46,47].

When this induced current is applied with adequate intensity to the cortex, it causes depolarization of cortical neuronal groups directly beneath the coil and in nearby and distant brain regions, leading to neurophysiological and behavioral outcomes [48,49]. This means that TMS, particularly when combined with EEG, can be a powerful tool for evaluating and modulating cortical excitability, plasticity, and functional connectivity. It allows for mapping specific brain regions’ roles in cognitive processes and investigating learning mechanisms. Moreover, the application of TMS can lead to a range of behavioral effects, including improved motor skill learning, altered cognitive control and decision-making, memory, language, and visuospatial abilities [48,49].

To stimulate activity in the human brain, the initial current usually needs to be around 4–8 kA with a rate of change peaking at 100–200 μs, which induces an electric current perpendicular to the coil’s surface and in the range of 7–15 mA/cm^2^ [50].

The strength of the induced current is directly proportional to the original current and decreases with distance due to factors such as bone, air, tissues, subdural and subarachnoid cerebrospinal fluid, and changes in the cortical structure [51].

Single-pulse Transcranial Magnetic Stimulation (spTMS) protocols involve the discharge of individual pulses, typically separated by intervals of 4–8 s. When a TMS pulse is applied to the primary motor cortex (M1), it can activate the corticospinal tract and related neural circuits, resulting in a muscle twitch in the area represented by the stimulated brain region [52].

This muscle twitch’s electrical activity can be recorded as a motor-evoked potential (MEP) through a surface electromyography (EMG) [53].

Higher cognitive functions, such as attention, memory, and language, can also be examined by applying TMS pulses to higher cortices and associated networks. This can temporarily disrupt physiological processes and/or behavioral activities supported by the stimulated brain regions [54,55]. The motor cortex is often the most targeted region in TMS studies because of its objective and easily obtainable neurophysiological output.

The TMS-induced MEP is often characterized by its amplitude and latency relative to the onset of spTMS, reflecting the functional integrity of the corticospinal tract. The MEP’s magnitude provides a measure of motor cortical excitability, which, in reality, reflects the balance between cortical excitation and inhibition, two opposing forces that control the activity in the cortex [56]. The MEP’s latency provides a measure of conduction time along central corticospinal motor pathways.

For all of its potential applications, TMS has been investigated as a potential tool for promoting recovery and neuroplasticity following brain injuries [57] and was used in DoC patients.

TBI can lead to a range of neurological symptoms, such as chronic pain [58], disturbances in mood [59] and sleep [60], and an increased risk of seizures and post-traumatic epilepsy [61]. These symptoms may be explained by a decrease in cortical inhibition, which results from a reduction in GABA-mediated synaptic inhibition and a decrease in GABA-synthesising enzymes at the cortical inhibitory synapses [62,63].

Research indicates that the impairment of cortical inhibition circuits in the motor regions of DoC patients can be assessed using TMS-EMG. A study conducted by Lapitskaya et al., which employed TMS-EMG on a group of 24 UWS/VS patients, 23 MCS patients, and 14 healthy controls, found that cortical motor regions for DoC patients were stimulated in an abnormal manner [64]; compared to the healthy control group, the patients exhibited higher Resting Motor Thresholds and lower average amplitudes of maximal peak-to-peak M-wave, MEP, Sensory Evoked Potential, and Short-latency Afferent Inhibition.

In another study, Bagnato and colleagues used paired-pulse TMS to detect significant alterations in the transmissions of inhibitory and excitatory neurons in UWS/VS patients [65]. Paired-pulse TMS (ppTMS) refers to the delivery of two magnetic pulses in rapid succession to the brain. The two pulses can be delivered with fixed or adaptive stimulus parameters to examine cortical excitability in the human motor cortex and probe intra-cortical and inter-cortical connections [66]. When compared to the healthy control group, the phenomena of Intracortical Inhibition (ICI) and Intracortical Facilitation in UWS/VS patients were significantly diminished [65].

However, studies using different ppTMS measures of ICI have reported mixed results, including facilitation, suppression, and no effect on MEPs following mild TBI [67].

In contrast to ppTMS, which involves delivering two magnetic pulses in rapid succession to the brain, other protocols utilize repetitive Transcranial Magnetic Stimulation (rTMS). These protocols involve delivering multiple pulses or bursts of stimulation at a fixed frequency ranging from 0.5 to 20 Hz. The duration of rTMS can vary from a few seconds to 30–40 min. This technique allows for the modulation of cortical excitability even beyond the stimulation period and can be applied to both motor and non-motor regions of the brain. As a result, it induces effects on brain activity that can be observed locally as well as in distant areas [68,69].

While high-frequency (≥5 Hz) rTMS protocols were found to generally increase cortical excitability as observed by MEPs [70], low-frequency (≤1 Hz) rTMS protocols were found to decrease it [70]. However, the impact of rTMS can also depend on the state of the cortex at the time of stimulation, which can be leveraged to enhance the specificity of the rTMS-induced effects [71].

Using rTMS, it is possible to depolarize neurons, alter the state of the cortex, and either excite or suppress the functionality of the local cerebral cortex between the stimulation coil and remote areas. As such, rTMS can be employed to amplify specific cognitive processes or modulate the activity of particular regions of the brain [72].

A study found that 60% of DoC patients began to show signs of visual tracking, emotional response, and even indicative action after receiving 5 Hz rTMS intervention, which corresponded to significant increases in the Glasgow Coma Scale and the CRS-R [73].

rTMS was also found to have a positive impact on the behavioral and resting-state functional connectivity of individuals with disorders of consciousness. The DoC patients who underwent 20 Hz rTMS treatment showed significant improvements in consciousness level, response to commands, and motor function compared to those receiving sham treatment. Moreover, resting-state fMRI analysis indicated that rTMS led to changes in functional connectivity patterns within the brain [74].

Another study involved 10 UWS/VS patients who received 10 Hz rTMS over the left dorsolateral prefrontal cortex (DLPFC). The findings indicated several positive outcomes from the procedure, including increased arousal and responsiveness, improved cognitive functions such as attention and working memory, and enhanced motor responses [75].

The effects of 10 Hz rTMS were also investigated by Xia and colleagues, who stimulated the left DLPFC and conducted quantitative EEG analysis in 18 chronic DoC patients. They found decreased low-frequency band power and increased high-frequency band power in the patient group, especially for those diagnosed with MCS, suggesting that quantitative EEG can be useful for assessing the effect of rTMS in DoC patients [76].

To study the therapeutic efficacy of rTMS, a randomized, double-blind, sham-controlled trial was conducted in 40 DoC patients with the administration of 20 Hz active-rTMS or sham-rTMS. It was found that compared to those treated by sham-rTMS, some patients showed significantly improved markers of consciousness. However, rTMS did not significantly enhance the awakening ratio [77].

Manganotti et al. explored 20 Hz rTMS impact on six MCS and UWS/VS patients observing significant behavioral and neurophysiological changes in only one MCS patient, correlating EEG reactivity and clinical response after rTMS [78]. Nonetheless, some research indicates that 20 Hz rTMS at M1 has no therapeutic impact on both MCS [74] and patients with UWS/VS [79], leaving the effectiveness of this approach unclear.

A recent study protocol has been proposed that will involve 30 DoC and investigate the potential use of 10 Hz rTMS in aiding the recovery of consciousness. The key goal is to apply stimulation over individualized target areas for each patient, which will be based on different areas of injury; this work presents an important step, as previous works focused on non-specific target areas for stimulation with low effectiveness for DoC patients [80].

In a study that involved 14 UWS/VS, 7 MCS, and healthy subjects, significant changes were found in TMS-evoked potentials and TMS-evoked connectivity in the healthy and MCS groups, but no significant changes were found in UWS/VS patients. These findings suggest that rTMS can effectively modulate effective connectivity in MCS patients [81].

Using TMS in conjunction with EEG offers an immediate approach to identifying the brain’s state and observing the dynamics of a wide range of cortical regions [79]. This non-intrusive method allows for the assessment of brain excitability, instant connectivity, and ephemeral brain states. When applied to the cerebral cortex, TMS elicits synaptic activity that can be viewed and quantified through EEG, a phenomenon known as TMS-evoked potentials (TEPs) [82].

Consciousness is supported by cortex–cortex and corticothalamic–cortex connections that also involve distant brain regions [83]. Since TMS-EEG allows for the assessment of effective connectivity, it was possible to differentiate between states of reduced awareness (e.g., sleep and anesthesia) and higher levels of consciousness (being awake or experiencing dreams) [84].

Previous findings showed that the breakdown of communication within the cerebral cortex during sleep may be necessary for consciousness. It was shown that in wakeful participants, TMS caused neural activity to spread across specific brain regions. However, during non-REM sleep, the TMS stimulus only generated neural activity at the stimulated site [85]. When studying this concept in DoC patients, Ragazzoni and colleagues found that in UWS/VS patients, TMS elicits a localized EEG response, indicating disrupted effective connectivity. In MCS patients, TMS often induces a more complex EEG activation pattern, but not at the same level as that of healthy individuals [86].

These studies emphasize the importance of the perturbational complexity index (PCI) as a marker of consciousness in TMS studies. Since consciousness relies on the brain’s ability to support complex activity patterns that are both integrated and information-rich, a measure that can capture this complexity is needed to measure the conscious level. When TMS is used to perturb the cortex, PCI measures the algorithmic complexity of the resulting electrocortical responses, which renders it a promising measure. Extensive testing on diverse subject populations, including healthy individuals in different conscious states and patients recovering from a coma, has shown that PCI can reliably discriminate levels of awareness. This measure may pose a solution to the challenge of subjectivity in assessing consciousness, as it provides an objective tool that can be employed at the bedside of patients with acquired brain injuries [87].

Casarotto and colleagues found that PCI was able to effectively stratify unresponsive DoC patients into different subgroups. EEG analysis of healthy subjects, MCS, and UWS/VS patients showed decreasing PCI values with increasing DoC severity [88].

In a study on DoC patients using 10 Hz rTMS applied to the DLPFC, a significant increase in the level of consciousness was observed, which was indicated by the CRS-R assessment, TEP, and PCI. These findings highlight the concurrent use of TMS-EEG and PCI as efficient tools for assessing the therapeutic effectiveness of rTMS in DoC patients [89].

Another study utilized the fast perturbational complexity index (PCIst) to differentiate between healthy individuals, MCS, and UWS/VS patients based on transcranial magnetic stimulation-evoked potentials (TMS-TEP). PCIst demonstrated significant differences in specific frequency bands, providing a potential tool for quantifying consciousness levels and assessing diagnosis and prognosis in DoC patients [90].

In a study that combined rTMS with standard rehabilitation on the recovery of consciousness in patients with persistent vegetative state, it was found that the group receiving rTMS showed significant improvements in the CRS-R and EEG grading indices following 30 and 60 days of treatment compared to the control group [91].

Finally, a pilot study evaluated the impact of rTMS, amantadine, and their combination on neurobehavioral gains in four DoC patients 1–15 years after their traumatic brain injury. Changes were observed in resting-state functional connectivity for language, salience, and sensorimotor networks after the period of treatment. This finding suggested that the combination of treatments that rely on different mechanisms to modulate neural activity may aid neurobehavioral recovery in DoC patients [92].

A study aims to uncover the neural responses of DoC patients to rTMS. Participants included DoC patients and healthy subjects who received rTMS. Measurements before and after rTMS were made with TMS-EMG. Notable changes in TMS-evoked potentials were observed in healthy participants but not in DoC patients. However, TMS-evoked connectivity increased significantly in healthy and MCS patients and was positively correlated with CRS-R scores. No significant changes were observed in UWS/VS patients [81].

The results of TMS studies are summarized in Table 2.

### 3.3. tDCS

While TMS can be a useful neurofeedback technique for DoC patients, another promising technique is transcranial direct current stimulation (tDCS), which offers a simple approach for localized brain stimulation. In contrast with TMS, tDCS does not induce direct neuronal action potentials because the static fields generated by tDCS do not provide the rapid depolarization required to produce action potentials in neural membranes [93]. Therefore, for tDCS to be effective, it is typically administered together with motor training tasks. The highest effects of tDCS are observed when it is applied before or during the task [94].

tDCS traces its origins back to the late 19th century, shortly after the advent of electricity [95]. It is a non-invasive approach that delivers a weak direct current (usually 1–2 mA) to specific cortical regions beneath two opposing electrodes contained in saline-soaked sponges [96]. It was found that anodal polarity (positive electrode) enhances neuronal activity, while cathodal polarity (negative electrode) reduces neuronal activity [97].

In more recent years, interest in tDCS was renewed under the guidance of Dr. Walter Paulus and his team in Gottingen, Germany, with over a hundred scientific articles published in the last decade [98]. The effects of tDCS on the brain are yet to be fully comprehended [93], but studies indicate that it can increase cortical excitability and improve memory in healthy individuals [99,100].

Depending on whether the area of focus is put into contact with an anode or cathode, researchers refer to the method as anodal or cathodal tDCS. The electrode placed in the target area is typically designated as the “active electrode”, while the other serves as the “reference electrode”. When both electrodes are positioned on conductive surfaces such as the scalp, a direct current passes between the anode and the cathode, resulting in distinct modifications in the excitability of the underlying cortical tissue. In this process, the cathode, which is negatively charged, serves as the exit point for the electric current. A smaller cathode can create a more concentrated delivery of charge to a specific brain region since more charge is aligned underneath the smaller exit point. Therefore, the size of the affected brain region can be influenced by modifying the size of the cathodal electrode (with a smaller size leading to more focused effects) [101] or by altering the size and position of the anodal electrode [102,103]. This stimulation technique can, therefore, be rather intricate as specific effects vary with different brain regions and the properties of the electrode used to deliver the current [104]. For instance, Accornero and colleagues noted that 10-min anodal tDCS decreased visual evoked response amplitudes, while 10-min cathodal tDCS increased them for a few minutes post-stimulation [105].

Regarding the duration of effects, changes induced by tDCS can persist for a considerable time post-treatment, although more precise estimates of durations continue to be investigated [106]. Nevertheless, alterations caused by tDCS can induce behavioral changes and even improve the neuroplasticity [107].

Numerous studies have demonstrated that this technique can alter perceptual, cognitive, and behavioral functions when applied to different brain regions. In addition, initial findings imply that tDCS could potentially have therapeutic benefits in the management of various brain disorders [93].

In a study involving ten chronic stroke patients [108], each participant underwent two separate sessions. They received either anodal tDCS or sham tDCS in a randomized order. To evaluate the brain’s response following tDCS, the team used spTMS. They found that when patients received anodal tDCS, those with stronger connections in a specific brain network showed a greater increase in brain responsiveness. This network included regions near the site of stimulation in the affected hemisphere’s motor cortex, as well as areas close to the affected hemisphere’s parietal cortex and the fronto-temporal cortex in the opposite hemisphere. These connections were observed in a specific frequency range (8–13 Hz) known as the alpha band. Importantly, this relationship between brain network connectivity and increased brain responsiveness was not seen after the placebo (sham) stimulation.

Bai et al. [109] observed that tDCS can effectively influence cortical excitability in DoC patients, and the changes in excitability, both temporally and spatially, differ between MCS and UWS/VS patients. In their study, which compared to baseline and sham stimulation, MCS patients demonstrated increased overall cerebral excitability in the initial time windows (0–100 ms and 100–200 ms). On the other hand, UWS/VS patients showed an increase in overall cerebral excitability in the 0–100 ms interval, but a decrease was witnessed in the 300–400 ms interval. This difference was likely due to differences in the severity of brain injury, which reflects varying degrees of connection integrity for different patients. These findings also suggest that the modulation effects of tDCS may be influenced by the underlying structural integrity of the cortical network in question [109].

Another study [110] explored the impact of tDCS applied to the left DLPFC on the behavior and brain activity of patients suffering from prolonged DoC. The authors adopted a double-blind, sham-controlled, crossover design in which thirteen patients with severe brain injuries were subjected to either an active or a sham tDCS session in a randomized order. Following the active tDCS session, an increase in relative power in both the alpha band in the central regions and the theta band in the frontal and posterior regions was observed. Behavioral enhancements were observed in three patients after the active tDCS session and in one patient after the sham session. The authors concluded that a single tDCS session could induce changes in brain activity in prolonged DoC patients, but these changes might not always be associated with notable behavioral advancements.

Another study focused on specific DoC pathology and investigated the potential of tDCS to enhance consciousness in patients diagnosed as UWS/VS and MCS [97]. The treatment included a week of sham tDCS and two weeks of real tDCS, both administered for 20 min daily for five days a week. Following treatment, all MCS patients demonstrated immediate clinical progress, while no such improvement was observed in UWS/VS patients. However, one UWS/VS patient, six years into the condition, exhibited improvement and transitioned to MCS at the one-year follow-up. The authors suggested that tDCS could be promising for treating severe consciousness disorders, with the severity and duration of the condition potentially influencing the effectiveness of the treatment.

Aloi et al. evidenced that many patients with prolonged DoC maintain high cognitive function and awareness [111], a phenomenon called cognitive-motor dissociation [112]. However, detecting covert cognition in these patients does not improve their prognosis, as most remain in a state of low responsiveness. In their work, they evidenced that tDCS could present a promising prospective therapeutic tool for MCS patients when used over several sessions. However, current results are inconsistent due to small study populations, diverse methodologies regarding tDCS parameters and outcome measures, and challenges associated with electrode placement and the heterogeneity of brain damage observed in these patients [111].

Thibaut and colleagues [113] studied the impact of left DLPFC tDCS on consciousness in patients with severe brain injuries at least one week after the acute event. Using a double-blind, sham-controlled crossover design, they delivered anodal and sham tDCS to patients in MCS or UWS/VS. They observed significant treatment effects on CRS-R scores in MCS patients but not in UWS/VS patients. Interestingly, 43% of MCS patients and 8% of UWS/VS patients showed signs of increased consciousness post-tDCS, which were not observed for pre-tDCS or sham evaluations. However, these tDCS-induced changes did not affect long-term outcomes. The conclusion was that tDCS can temporarily enhance signs of consciousness in MCS patients, as reflected in CRS-R score changes.

In a study by Hermann and colleagues [114], 60 DoC patients were stimulated via the left DLPFC. Results showed a 20% improvement in clinical behavioral assessment. In the EEG analysis, the improvements were correlated with increased power and long-range cortico-cortical functional connectivity.

A retrospective study evaluated resting-state functional MRI of 16 MCS patients who received a single left DLPFC tDCS in a randomized trial. Results indicated that six tDCS responders showed increased intra-network connectivity, particularly with the left inferior frontal gyrus; differently, non-responders displayed increased connectivity between left DLPFC and midline cortical structures [115].

The results of tDCS studies are summarized in Table 3.

## 4. Discussion

In this work, we provided an overview of how BCI, TMS, and tDCS are promising non-invasive techniques for managing DoC. However, they each face unique limitations that need addressing to maximize their potential.

BCI technology allows for direct communication between the brain and digital devices and is, hence, a promising tool for DoC patients. By translating brain-generated electrical signals into actionable commands, BCIs can facilitate responses to queries, item selection on screens, or control of devices, including wheelchairs [25].

Research has demonstrated potential uses for BCI in patients with minimal consciousness. For example, Coyle et al. [27] showed that EEG evaluations of unique sensorimotor patterns could allow for the use of BCI communication systems in patients unable to voluntarily control their movements. Moreover, Li and colleagues [28] examined the integration of multiple brain signals or varied BCI paradigms to improve performance, reliability, and robustness in identifying number processing and mental arithmetic in DOC patients. Xiao and colleagues [30] also applied BCI to evaluate visual fixation in DoC patients, demonstrating its diagnostic potential. In another significant study, Pan et al. [31] identified patients with CMD, a condition where patients exhibit no observable response to commands, providing evidence of the direct impact of BCI on consciousness.

Studies using gaze-independent audiovisual BCI systems, such as the one by Xie et al. [32] assessed the capability of DoC patients to pay attention to sequential visual stimuli, further showcasing BCI’s potential in detecting patient awareness. Meanwhile, Lulé et al. [33] proved the possibility of detecting command following and functional communication in DoC patients via EEG-based BCI.

TMS offers another promising approach to examining and treating DoC. It allows for non-invasive modulation of brain activity and can facilitate neuroplasticity, potentially aiding in recovery [73,74,77,80]. Combining TMS with EEG further enhances its utility, allowing for a more nuanced understanding of brain dynamics in the DoC [79]. In particular, the PCI derived from TMS-EEG data offers a quantitative measure of consciousness, facilitating differentiation between healthy individuals, MCS, and UWS/VS patients [87,88].

Finally, tDCS presents as an additional non-invasive technique for brain modulation and holds potential therapeutic benefits for various brain disorders, including the DoC [93,95]. It facilitates targeted cortical stimulation, with studies indicating improved memory in healthy individuals [99,100] and potential modulatory effects in DoC patients [109,110]. For instance, evidence suggests changes in cortical excitability in DoC patients after tDCS [116], possibly influenced by the structural integrity of the cortical network [109]. Furthermore, there are indications of tDCS-induced enhancement of consciousness in MCS patients and, to a lesser extent, UWS/VS patients [97,113]. It is also associated with observed behavioral improvements and neuroplasticity enhancements [107].

While BCI, TMS, and tDCS each have their own advantages, they also have unique limitations that need addressing to maximize their potential and allow for further clinical implementations.

The limitations of BCI are considerable. For instance, BCI performance may vary between patients due to individual neurophysiological differences, thus affecting the system’s accuracy and reliability [68,69]. Furthermore, signal acquisition from severely damaged brains poses challenges in terms of signal quality, with complex signal processing and artifact rejection required [70]. In fact, in the context of DoC, the processing and analysis of neurofeedback signals present unique challenges. A significant factor is the inherent variability of brain activity in DoC conditions, which can be influenced by the extent and location of brain damage, the patient’s level of consciousness, and other individual characteristics. This variability can affect the quality and interpretability of the signals obtained from such neurofeedback tools as EEG, complicating their analysis and potentially limiting their usefulness in guiding interventions. Specifically, brain damage in DoC conditions may result in altered or abnormal EEG patterns, including slow-wave activity, epileptiform discharges, and other irregularities. These abnormal signals can mask or distort the signals of interest in neurofeedback applications, such as those associated with cognition or sensory processing. Traditional signal-processing techniques may not be sufficient to capture the complex and subtle changes in brain activity that could indicate changes in a patient’s level of consciousness. Advanced analysis methods, such as machine learning algorithms, could potentially improve the interpretation of these signals, but their implementation is non-trivial and requires further research and validation.

The application of BCI also raises ethical concerns, primarily surrounding patient autonomy and the accuracy of patient representations [71].

TMS, on the other hand, can modulate neuronal activity and provide diagnostic and therapeutic benefits for DoC patients [75]. Despite these advantages, TMS comes with its own set of limitations. Its stimulation depth is limited, thus not adequately reaching deep brain structures. Moreover, the technology cannot directly trigger neuronal action potentials in patients with severe brain injuries. Hence, for TMS to be effective, it often needs to be paired with concurrent behavioral tasks [91]. Another challenge is the discomfort or potential side effects such as headaches or seizures, though these are rare [81]. Moreover, individual differences in neuroanatomy may lead to variable responses to TMS, limiting its reproducibility [82].

The tDCS presents as a compelling method for localized brain stimulation, but it also has important limitations. Although capable of modifying cortical excitability and potentially improving consciousness in DoC patients [97,109], it does not induce direct neuronal action potentials. Thus, to be effective, it often has to be administered along with motor training tasks [93,94]. Further, the impacts are variable depending on the different brain regions, their unique characteristics, and the patient’s individual neurophysiological state [104]. The understanding of tDCS’s long-term effects and outcomes remains limited, requiring further exploration [106]. The heterogeneity in brain damage among DoC patients contributes to inconsistent results [111].

Moreover, while NF tools hold promise for treating DoC, their effectiveness in patients with UWS/VS appears to be limited. There could be several reasons for this, such as a lack of responsiveness and brain damage. It is important to consider that the primary characteristic of UWS/VS is a lack of responsiveness. While these patients may open their eyes and exhibit reflexive behaviors, they do not demonstrate any meaningful responses to stimuli. This lack of responsiveness might limit the efficacy of NF tools, which rely on the ability of the patient to respond to feedback and adjust their brain activity accordingly. Moreover, depending on the cause and severity of the UWS/VS, substantial brain damage may inhibit the effectiveness of NF tools. Furthermore, NF tools largely depend on the accuracy of real-time monitoring of brain activity, often through EEG. However, in severe cases of UWS/VS, the brain’s electrical activity may be too impaired or erratic to be accurately measured and used for effective NF.

There are some potential directions to improve the effectiveness of NF tools in treating UWS/VS. Technological advancements could lead to more accurate and sensitive tools for monitoring brain activity, potentially increasing the effectiveness of NF in UWS/VS patients. In addition, improvements in machine learning algorithms could lead to more personalized and effective treatment strategies. NF might be more effective when combined with other treatments. For instance, it could be used in conjunction with traditional rehabilitation therapies, pharmacological treatments, or other neuromodulation techniques. These considerations highlight that further research is needed to better understand UWS/VS and how it affects brain function. This could lead to the development of more effective NF techniques tailored specifically for these patients.

## 5. Conclusions

We conclude by emphasizing the importance of further research and therapeutic developments for patients suffering from acquired brain injuries and DoC. Despite their limitations, BCI, TMS, and tDCS present promising neurofeedback tools that can assist in the diagnosis and care of these patients. While no single “gold-standard” treatment exists, the heterogeneity of DoC conditions calls for personalized therapeutic approaches depending on the level of severity and etiology of brain damage. Accordingly, the existence of several tools, each with its own strengths and weaknesses, can be leveraged to address individual differences among DoC patients. Ultimately, the goal of the research we reviewed is to establish future clinical guidelines and standards for treating severe neurological disorders. To meet this goal, different neurofeedback tools require further research to understand their most appropriate applications, which will help overcome the limitations we discussed [68,82,111]. 

## Figures and Tables

**Table 1:** Studies on BCI and Applications in Disorders of Consciousness.

Author(s)	Reference	Patients	Study Description	Main Findings
Coyle et al.	[27]	4 MCS	Assessed awareness in MCS patients using an EEG-based BCI, evaluating sensorimotor rhythm modulation with visual and auditory feedback.	MCS patients demonstrated significant brain activation in the initial assessment. They received real-time feedback to enhance arousal and were able to operate a basic BCI communication system despite a lack of motor responses.
Li et al.	[28]	3 MCS, 6 UWS/VS, 2 EMCS	Used a combined P300 and Steady-State Visual Evoked Potential (SSVEP) BCI system for number processing and mental maths tasks.	2 UWS/VS, 1 MCS and 2 EMCS patients performed above chance, suggesting covert cognition.
Xiao et al.	[30]	15 DoC	The BCI system was used to assist the visual fixation assessment of DOC patients.	1 patient did not show visual fixation in the CRS-R assessment but achieved a significant level of accuracy in the BCI assessment.
Pan et al.	[31]	45 UWS/VS, 33 MCS	Motor imagery BCI was used to identify cognitive-motor dissociation.	18 UWS/VS and 16 MCS showed a dissociation between BCI and behavioral responses.
Xie et al.	[32]	8 DoC	Gaze-independent audiovisual BCI system.	3 patients demonstrated command following and number recognition.
Lule et al.	[33]	2 LIS, 13 MCS, 3 UWS/VS	BCI was used to detect consciousness in DOC patients by assessing their response to command and communication.	Detected command following in 1 MCS and 1 LIS patient.
Xiao	[35]	10 UWS/VS, 8 MCS	A novel audiovisual BCI system was developed to simulate sound localization evaluation in CRS-R	All patients showing sound localization in the CRS-R were among those detected by BCI

**Table 2:** Studies on TMS and Applications in Disorders of Consciousness.

Author(s)	Reference	Patients	Study Description	Main Findings
Lapitskaya et al.	[64]	24 UWS/VS, 23 MCS, 14 healthy controls	To assess corticospinal excitability using single and paired-pulse TMS over M1 while recording MEPs with EMG, and to compare motor thresholds and MEP amplitudes between groups.	UWS/VS and MCS showed increased motor thresholds and reduced MEP amplitudes compared to healthy controls.
Bagnato et al.	[65]	5 UWS/VS, 10 healthy controls	To evaluate the inhibitory and excitatory interneuronal circuits in patient UWS/VS using ppTMS following a traumatic brain injury.	UWS/VS patients showed reduced intracortical inhibition compared to healthy controls.
Xie et al.	[73]	11 UWS/VS, 7 MCS, 2 coma	Ten patients received 28 sessions of rTMS treatment with 5 Hz on the right dorsolateral prefrontal cortex (DLPFC) in addition to conventional therapy.	In 60% of patients, GCS and CRS-R scores were significantly increased after rTMS
Naro et al.	[75]	10 UWS/VS, 10 healthy controls	An amount of 10 Hz rTMS over left DLPFC in daily sessions for 2 weeks. CRS-R measured before and after.	Patients showed increased arousal, cognition, and motor responses after rTMS per CRS-R.
Xia et al.	[76]	18 DoC (9 UWS/VS, 9 MCS)	An amount of 10 Hz rTMS over left DLPFC for 10 min. EEG was recorded before and multiple times after stimulation.	Decreased delta/theta waves and increased alpha/beta waves post-rTMS, especially in MCS patients.
Fan et al.	[77]	40 DoC	To investigate the therapeutic efficacy of rTMS in patients with disorders of consciousness. CRS-R compared to baseline. A 20-Hz active TMS on left DLPFC and sham-rTMS protocol.	Some patients showed significant CRS-R score increases compared to sham rTMS.
Manganotti et al.	[78]	3 MCS, 3 UWS/VS	To investigate the reactivity of EEG and the clinical response in six severely DoC by single session of 20-Hz rTMS over the motor cortex	One MCS patient showed neurophysiological and clinical changes. No effects seen in UWS/VS.
Cincotta et al.	[79]	11 UWS/VS patients	An amount of 20 Hz rTMS over M1 daily for 5 days. CRS-R measured before and after.	No significant effects on consciousness observed based on CRS-R scores.
Xu et al.	[80]	30 DoC	An amount of 10 Hz rTMS over individualized brain regions, double-blind crossover randomized sham-controlled trial.	Study protocol, results pending.
Xia et al.	[81]	14 UWS/VS, 7 MCS, 14 healthy subjects	TMS-EEG responses were recorded and compared between groups.	TMS-evoked potentials and connectivity changed in MCS but not UWS/VS.
Ragazzoni et al.	[86]	8 UWS/VS, 5 MCS, 5 healthy subjects	To improve the accuracy of diagnosing the differences between UWS/VS and MCS by assessing cortical reactivity and effective connectivity using TMS combined with EEG (cortical potentials evoked by TMS (TEPs)), TMS was applied to the less-affected hemisphere of patients and the dominant hemisphere of controls, targeting the primary motor cortex. A total of 400 TMS pulses (200 real, 200 sham) were delivered at random intervals between 0.25 to 0.5 Hz during a 60-min session conducted at the patients’ bedside.	TEP results suggest that cortical reactivity and connectivity are severely impaired in all UWS/VS patients, whereas in most MCS patients; the TEPs are preserved but with abnormal features.
Casarotto et al.	[88]	38 MCS, 43 UWS/VS, 150 healthy subjects	To stratify unresponsive patients, such as those in a vegetative state or minimally conscious state, using an independently validated index of brain complexity, single TMS pulses were delivered with a focal biphasic stimulator. TMS targets were selected bilaterally within the middle–caudal portion of the superior frontal gyrus and within the superior parietal lobule	PCI values were significantly lower in patients with disorders of consciousness.
Bai et al.	[89]	1 DOC	TMS-EEG to assess effects of rTMS on brain modulation of DOC. Twenty sessions of 10 Hz rTMS were applied over the DLPFC.	By the CRS-R, TEP, and PCI, a significant increase in the level of consciousness was observed.
Wang et al.	[90]	30 healthy subjects, 76 MCS, 105 UWS/VS	PCIst was used to the diagnosis and prognosis of DOC patients. The PCIst was used to assess the time-space complexity of TMS-evoked potentials (TEP).	PCIst demonstrated significant differences in specific frequency bands between groups.
Zhang et al.	[91]	48 DoC	To explore the effect of combining rTMS and conventional rehabilitation on the recovery of consciousness in patients in a persistent vegetative state (PVS).	The group receiving rTMS showed significant improvements in the CRS-R and EEG grading indices
Pape et al.	[92]	4 DoC	To evaluate the impact of repetitive transcranial magnetic stimulation (rTMS), amantadine (AMA), and their combination.	Auditory-language gains were observed after rTMS, which increased when rTMS preceded rTMS + AMA.

**Table 3:** Studies on tDCS and Applications in Disorders of Consciousness.

Author(s)	Reference	Patients	Study Description	Main Findings
Angelakis et al.	[97]	10 DoC	A 20-min anodal tDCS was applied over the left DLPFC at 2 mA intensity, 5 days per week for 2 weeks. Assessed behavior with CRS-R.	Clinical improvement was observed in all MCS patients. One UWS/VS patient improved to MCS at 1-year follow-up.
Hordacre et al.	[108]	10 chronic stroke	Participants were randomized to initially receive either anodal or sham tDCS to the lesioned primary motor cortex (M1). Single-pulse TMS over lesioned M1 before and after tDCS	An enhanced motor cortical connectivity was observed related to increased excitability after a single session of anodal tDCS.
Bai et al.	[109]	10 MCS, 10 UWS/VS	A 20-min anodal tDCS over left DLPFC at 2 mA intensity. EEG recorded before, during, and after stimulation. Assessed global changes in cortical excitability.	MCS patients showed increased cortical excitability after tDCS. UWS/VS patients had more variable responses, with differences in excitability changes over time.
Carriere et al.	[110]	13 DoC	A 20-min anodal tDCS over left DLPFC at 2 mA intensity. High-density EEG recorded before and after stimulation. CRS-R was administered before and after to assess behavioral changes.	Increased alpha and theta EEG power observed after tDCS. Three patients also showed behavioral improvements according to CRS-R scores.
Thibaut et al.	[113]	30 MCS, 25 UWS/VS	A 20-min anodal tDCS over left DLPFC. CRS-R was administered before and after stimulation.	CRS-R scores increased in 43% of MCS patients and 8% of UWS/VS patients after tDCS.
Hermann et al.	[114]	60 DoC	A 20-min anodal tDCS over left DLPFC at 2 mA intensity. High-density EEG and CRS-R were administered before and after stimulation.	A total of 20% of patients showed improved CRS-R scores and EEG functional connectivity after tDCS.
Cavaliere et al.	[115]	16 MCS	A 20-min anodal tDCS over left DLPFC at 2 mA intensity. Resting-state fMRI was performed before and after stimulation.	tDCS responders showed increased functional connectivity in motor networks compared to non-responders.

## Data Availability

Not applicable.

## Abbreviations

EEG	Electroencephalography
BCI	Brain–Computer Interface
tDCS	Transcranial direct current stimulation
TMS	Transcranial Magnetic Stimulation
DoC	Disorder of Consciousness
ABI	Acquired Brain Injury
UWS/VS	Unresponsive Wakefulness Syndrome/Vegetative State
MCS	Minimally Conscious State
CRS-R	Coma Recovery Scale Revised
TBI	Traumatic Brain Injury
LOS	Length of Stay
NF	Neurofeedback
SSVEPs	Steady-state visual evoked potentials
CMD	Cognitive motor dissociation
ERPs	Evoke event-related potentials
LIS	Locked-in syndrome
spTMS	Single-pulse Transcranial Magnetic Stimulation
M1	Primary motor cortex
MEP	Motor-evoked potential
EMG	Electromyography
ppTMS	Paired-pulse TMS
ICI	Intracortical Inhibition
rTMS	Repetitive Transcranial Magnetic Stimulation
DLPFC	Dorsolateral prefrontal cortex
TEPs	TMS-evoked potentials
PCI	Perturbational complexity index

## References

[1] Kamalakannan S.K., Gudlavalleti A.S., Gudlavalleti V.S.M., Goenka S., Kuper H. (2015). Challenges in understanding the epidemiology of acquired brain injury in India. Ann. Indian Acad. Neurol..

[2] Suchy Y. (2023). Neuropsychological Interviewing of Adults.

[3] Teasell R., Bayona N., Marshall S., Cullen N., Bayley M., Chundamala J., Villamere J., Mackie D., Rees L., Hartridge C. (2007). A systematic review of the rehabilitation of moderate to severe acquired brain injuries. Brain Inj..

[4] Schnakers C. (2020). Update on diagnosis in disorders of consciousness. Expert Rev. Neurother..

[5] Bruno M.-A., Majerus S., Boly M., Vanhaudenhuyse A., Schnakers C., Gosseries O., Boveroux P., Kirsch M., Demertzi A., Bernard C. (2011). Functional neuroanatomy underlying the clinical subcategorization of minimally conscious state patients. J. Neurol..

[6] Giacino J.T., Fins J.J., Laureys S., Schiff N.D. (2014). Disorders of consciousness after acquired brain injury: The state of the science. Nat. Rev. Neurol..

[7] Giacino J.T., Katz D.I., Schiff N.D., Whyte J., Ashman E.J., Ashwal S., Barbano R., Hammond F.M., Laureys S., Ling G.S. (2018). Practice Guideline Update Recommendations Summary: Disorders of Consciousness. Arch. Phys. Med. Rehabil..

[8] Giacino J.T., Kalmar K., Whyte J. (2004). The JFK Coma Recovery Scale-Revised: Measurement characteristics and diagnostic utility. Arch. Phys. Med. Rehabil..

[9] Oberholzer M., Müri R.M. (2019). Neurorehabilitation of Traumatic Brain Injury (TBI): A Clinical Review. Med Sci..

[10] Bayona N.A., Bitensky J., Teasell R. (2005). Plasticity and Reorganization of the Uninjured Brain. Top. Stroke Rehabil..

[11] Andelic N., Bautz-Holter E., Ronning P., Olafsen K., Sigurdardottir S., Schanke A.-K., Sveen U., Tornas S., Sandhaug M., Roe C. (2012). Does an Early Onset and Continuous Chain of Rehabilitation Improve the Long-Term Functional Outcome of Patients with Severe Traumatic Brain Injury?. J. Neurotrauma.

[12] Turner-Stokes L., Pick A., Nair A., Disler P.B., Wade D.T. (2015). Multi-disciplinary rehabilitation for acquired brain injury in adults of working age. Cochrane Database Syst. Rev..

[13] Turner-Stokes L. (2008). Evidence for the effectiveness of multi-disciplinary rehabilitation following acquired brain injury: A synthesis of two systematic approaches. J. Rehabil. Med..

[14] Zhu X.L., Poon W.S., Chan C.C.H., Chan S.S.H. (2007). Does intensive rehabilitation improve the functional outcome of patients with traumatic brain injury (TBI)? A randomized controlled trial. Brain Inj..

[15] Hart T., Whyte J., Poulsen I., Kristensen K.S., Nordenbo A.M., Chervoneva I., Vaccaro M.J. (2016). How Do Intensity and Duration of Rehabilitation Services Affect Outcomes From Severe Traumatic Brain Injury? A Natural Experiment Comparing Health Care Delivery Systems in 2 Developed Nations. Arch. Phys. Med. Rehabil..

[16] Slade A., Tennant A., Chamberlain M.A. (2002). A randomised controlled trial to determine the effect of intensity of therapy upon length of stay in a neurological rehabilitation setting. J. Rehabil. Med..

[17] Formisano R., Contrada M., Aloisi M., Buzzi M.G., Cicinelli P., Della Vedova C., Laurenza L., Matteis M., Spanedda F., Vinicola V. (2017). Improvement rate of patients with severe brain injury during post-acute intensive rehabilitation. Neurol. Sci. Seve.

[18] Turner-Stokes L. (2007). Cost-efficiency of longer-stay rehabilitation programmes: Can they provide value for money?. Brain Inj..

[19] Jahanian Najafabadi A., Oh H., Imani H., Godde B. (2021). Effect of Neurofeedback Training Combined with Transcranial Direct Current Stimulation on Primary Insomnia.

[20] Maggio M.G., Naro A., La Rosa G., Cambria A., Lauria P., Billeri L., Latella D., Manuli A., Calabrò R.S. (2020). Virtual Reality Based Cognitive Rehabilitation in Minimally Conscious State: A Case Report with EEG Findings and Systematic Literature Review. Brain Sci..

[21] Wyckoff S., Birbaumer N. (2014). Neurofeedback and Brain-Computer Interfaces. Int. Rev. Neurobiol..

[22] Loriette C., Ziane C., Ben Hamed S. (2021). Neurofeedback for cognitive enhancement and intervention and brain plasticity. Rev. Neurol..

[23] Morlet D., Fischer C. (2013). MMN and Novelty P3 in Coma and Other Altered States of Consciousness: A Review. Brain Topogr..

[24] Sokoliuk R., Degano G., Banellis L., Melloni L., Hayton T., Sturman S., Veenith T., Yakoub K.M., Belli A., Noppeney U. (2020). Covert Speech Comprehension Predicts Recovery From Acute Unresponsive States. Ann. Neurol..

[25] Ramadan R.A., Refat S., Elshahed M.A., Ali R.A. (2014). Basics of Brain Computer Interface.

[26] Pichiorri F., Mattia D. (2020). Brain-computer interfaces in neurologic rehabilitation practice. Handb. Clin. Neurol..

[27] Coyle D., Stow J., McCreadie K., McElligott J., Carroll Á. (2015). Sensorimotor Modulation Assessment and Brain-Computer Interface Training in Disorders of Consciousness. Arch. Phys. Med. Rehabil..

[28] Li Y., Pan J., He Y., Wang F., Laureys S., Xie Q., Yu R. (2015). Detecting number processing and mental calculation in patients with disorders of consciousness using a hybrid brain-computer interface system. BMC Neurol..

[29] Pfurtscheller G., Allison B., Bauernfeind G., Brunner C., Solis Escalante T., Scherer R., Zander T., Mueller-Putz G., Neu-per C., Birbaumer N. (2010). The Hybrid BCI. Front. Neurosci..

[30] Xiao J., Pan J., He Y., Xie Q., Yu T., Huang H., Lv W., Zhang J., Yu R., Li Y. (2018). Visual Fixation Assessment in Patients with Disorders of Consciousness Based on Brain-Computer Interface. Neurosci. Bull..

[31] Pan J., Xie Q., Qin P., Chen Y., He Y., Huang H., Wang F., Ni X., Cichocki A., Yu R. (2020). Prognosis for patients with cognitive motor dissociation identified by brain-computer interface. Brain.

[32] Xie Q., Pan J., Chen Y., He Y., Ni X., Zhang J., Wang F., Li Y., Yu R. (2018). A gaze-independent audiovisual brain-computer Interface for detecting awareness of patients with disorders of consciousness. BMC Neurol..

[33] Lulé D., Noirhomme Q., Kleih S.C., Chatelle C., Halder S., Demertzi A., Bruno M.-A., Gosseries O., Vanhaudenhuyse A., Schnakers C. (2013). Probing command following in patients with disorders of consciousness using a brain-computer interface. Clin. Neurophysiol..

[34] Pokorny C., Klobassa D.S., Pichler G., Erlbeck H., Real R.G.L., Kubler A., Lesenfants D., Habbal D., Noirhomme Q., Risetti M. (2013). The Auditory P300-Based Single-Switch BCI: Paradigm Transition from Healthy Subjects to Minimally Conscious Patients. Artif. Intell. Med..

[35] Xiao J., He Y., Yu T., Pan J., Xie Q., Cao C., Zheng H., Huang W., Gu Z., Yu Z. (2022). Toward Assessment of Sound Locaization in Disorders of Consciousness Using a Hybrid Audiovisual Brain–Computer Interface. IEEE Trans. Neural Syst. Rehabil. Eng..

[36] Xiao J., Xie Q., Lin Q., Yu T., Yu R., Li Y. (2018). Assessment of Visual Pursuit in Patients With Disorders of Consciousness Based on a Brain-Computer Interface. IEEE Trans. Neural Syst. Rehabil. Eng..

[37] Allison B.Z., Cho W., Ortner R., Heilinger A., Edlinger G., Guger C., Schmorrow D.D., Fidopiastis C.M. (2017). Validation of a Brain-Computer Interface (BCI) System Designed for Patients with Disorders of Consciousness (DOC): Regular and Sham Testing with Healthy Participants. Proceedings of the Augmented Cognition, Enhancing Cognition and Behavior in Complex Human Environments.

[38] Spataro R., Heilinger A., Allison B., De Cicco D., Marchese S., Gregoretti C., La Bella V., Guger C. (2018). Preserved somatosensory discrimination predicts consciousness recovery in unresponsive wakefulness syndrome. Clin. Neurophysiol..

[39] Murovec N., Heilinger A., Xu R., Ortner R., Spataro R., La Bella V., Miao Y., Jin J., Chatelle C., Laureys S. (2020). Effects of a Vibro-Tactile P300 Based Brain-Computer Interface on the Coma Recovery Scale-Revised in Patients With Disorders of Consciousness. Front. Neurosci..

[40] Chatelle C., Spencer C.A., Cash S.S., Hochberg L.R., Edlow B.L. (2018). Feasibility of an EEG-based brain-computer interface in the intensive care unit. Clin. Neurophysiol..

[41] Huang J., Qiu L., Lin Q., Xiao J., Huang Y., Huang H., Zhou X., Shi X., Wang F., He Y. (2021). Hybrid asynchronous brain–computer interface for yes/no communication in patients with disorders of consciousness. J. Neural Eng..

[42] Eliseyev A., Gonzales I.J., Le A., Doyle K., Egbebike J., Velazquez A., Agarwal S., Roh D., Park S., Connolly E.S. (2021). De-velopment of a Brain-Computer Interface for Patients in the Critical Care Setting. PLoS ONE.

[43] Barker A., Jalinous R., Freeston I. (1985). Non-invasive magnetic stimulation of human motor cortex. Lancet.

[44] Rossi S., Antal A., Bestmann S., Bikson M., Brewer C., Brockmöller J., Carpenter L.L., Cincotta M., Chen R., Daskalakis J.D. (2021). Safety and recommendations for TMS use in healthy subjects and patient populations, with updates on training, ethical and regulatory issues: Expert Guidelines. Clin. Neurophysiol..

[45] Wagner T., Valero-Cabre A., Pascual-Leone A. (2007). Noninvasive Human Brain Stimulation. Annu. Rev. Biomed. Eng..

[46] Barker A.T. (1991). An Introduction to the Basic Principles of Magnetic Nerve Stimulation. J. Clin. Neurophysiol..

[47] Barker A. (2017). Transcranial Magnetic Stimulation—Past, present and future. Brain Stimul..

[48] Hallett M. (2007). Transcranial Magnetic Stimulation: A Primer. Neuron.

[49] Valero-Cabré A., Amengual J.L., Stengel C., Pascual-Leone A., Coubard O.A. (2017). Transcranial magnetic stimulation in basic and clinical neuroscience: A comprehensive review of fundamental principles and novel insights. Neurosci. Biobehav. Rev..

[50] Nahas Z. (2002). Handbook of Transcranial Magnetic Stimulation. J. Psychiatry Neurosci..

[51] Wagner T., Rushmore J., Eden U., Valero-Cabre A. (2009). Biophysical foundations underlying TMS: Setting the stage for an effective use of neurostimulation in the cognitive neurosciences. Cortex.

[52] Amassian V.E., Stewart M., Quirk G.J., Rosenthal J.L. (1987). Physiological Basis of Motor Effects of a Transient Stimulus to Cere-bral Cortex. Neurosurgery.

[53] Amassian V., Quirk G.J., Stewart M. (1990). A comparison of corticospinal activation by magnetic coil and electrical stimulation of monkey motor cortex. Electroencephalogr. Clin. Neurophysiol. Potentials Sect..

[54] Ganis G., Keenan J.P., Kosslyn S.M., Pascual-Leone A. (2000). Transcranial magnetic stimulation of primary motor cortex affects mental rotation. Cereb. Cortex.

[55] Mottaghy F.M., Pascual-Leone A., Kemna L.J., Töpper R., Herzog H., Müller-Gärtner H.-W., Krause B.J. (2003). Modulation of a brain–behavior relationship in verbal working memory by rTMS. Cogn. Brain Res..

[56] Noda Y., Barr M.S., Zomorrodi R., Cash R.F.H., Lioumis P., Chen R., Daskalakis Z.J., Blumberger D.M. (2021). Single-Pulse Transcranial Magnetic Stimulation-Evoked Potential Amplitudes and Latencies in the Motor and Dorsolateral Prefrontal Cortex among Young, Older Healthy Participants, and Schizophrenia Patients. J. Pers. Med..

[57] Bashir S., Mizrahi I., Weaver K., Fregni F., Pascual-Leone A., Ba I.M., Ba K.W. (2010). Assessment and Modulation of Neural Plasticity in Rehabilitation With Transcranial Magnetic Stimulation. PM&R.

[58] Andary M.T., Crewe N., Ganzel S.K., Haines-Pepi C., Kulkarni M.R., Stanton D.F., Thompson A., Yosef M. (1997). Traumatic Brain Injury/Chronic Pain Syndrome: A Case Comparison Study. Clin. J. Pain.

[59] Jorge R.E., Acion L., Starkstein S.E., Magnotta V. (2007). Hippocampal Volume and Mood Disorders After Traumatic Brain Injury. Biol. Psychiatry.

[60] Hou L., Han X., Sheng P., Tong W., Li Z., Xu D., Yu M., Huang L., Zhao Z., Lu Y. (2013). Risk Factors Associated with Sleep Disturbance following Traumatic Brain Injury: Clinical Findings and Questionnaire Based Study. PLoS ONE.

[61] Lucke-Wold B.P., Nguyen L., Turner R.C., Logsdon A.F., Chen Y.-W., Smith K.E., Huber J.D., Matsumoto R., Rosen C.L., Tucker E.S. (2015). Traumatic brain injury and epilepsy: Underlying mechanisms leading to seizure. Seizure.

[62] Huusko N., Pitkänen A. (2014). Parvalbumin immunoreactivity and expression of GABAA receptor subunits in the thalamus after experimental TBI. Neuroscience.

[63] Traumatic Brain Injury Increases Cortical Glutamate Network Activity by Compromising GABAergic Control | Cerebral Cortex | Oxford Academic. https://academic.oup.com/cercor/article/25/8/2306/313160.

[64] Lapitskaya N., Gosseries O., De Pasqua V., Pedersen A.R., Nielsen J.F., de Noordhout A.M., Laureys S. (2013). Abnormal Corticospinal Excitability in Patients with Disorders of Consciousness. Brain Stimul..

[65] Bagnato S., Boccagni C., Sant’angelo A., Prestandrea C., Rizzo S., Galardi G. (2012). Patients in a vegetative state following traumatic brain injury display a reduced intracortical modulation. Clin. Neurophysiol..

[66] Amandusson Å., Flink R., Axelson H. (2017). Comparison between adaptive and fixed stimulus paired-pulse transcranial magnetic stimulation (ppTMS) in normal subjects. Clin. Neurophysiol. Pract..

[67] Opie G.M., Foo N., Killington M., Ridding M.C., Semmler J.G. (2019). Transcranial Magnetic Stimulation-Electroencephalography Measures of Cortical Neuroplasticity Are Altered after Mild Traumatic Brain Injury. J. Neurotrauma.

[68] Pascual-Leone A., Gomez-Tortosa E., Grafman J., Alway D., Nichelli P., Hallett M. (1994). Induction of visual extinction by rapid-rate transcranial magnetic stimulation of parietal lobe. Neurology.

[69] Rotenberg A., Horvath J.C., Pascual-Leone A. (2014). The Transcranial Magnetic Stimulation (TMS) Device and Foundational Techniques. Neuromethods.

[70] Fitzgerald P., Fountain S., Daskalakis Z. (2006). A comprehensive review of the effects of rTMS on motor cortical excitability and inhibition. Clin. Neurophysiol..

[71] Silvanto J., Cattaneo Z., Battelli L., Pascual-Leone A. (2008). Baseline Cortical Excitability Determines Whether TMS Disrupts or Facilitates Behavior. J. Neurophysiol..

[72] Harris-Love M.L., Cohen L.G. (2006). Noninvasive Cortical Stimulation in Neurorehabilitation: A Review. Arch. Phys. Med. Rehabil..

[73] Xie Y., Zhang T., Chen A.C. (2015). Repetitive Transcranial Magnetic Stimulation for the Recovery of Stroke Patients with Dis-turbance of Consciousness. Brain Stimul. Basic Transl. Clin. Res. Neuromodulation.

[74] Liu X., Meng F., Gao J., Zhang L., Zhou Z., Pan G., Luo B. (2018). Behavioral and Resting State Functional Connectivity Effects of High Frequency rTMS on Disorders of Consciousness: A Sham-Controlled Study. Front. Neurol..

[75] Naro A., Russo M., Leo A., Bramanti P., Quartarone A., Calabrò R.S. (2015). A Single Session of Repetitive Transcranial Magnetic Stimulation over the Dorsolateral Prefrontal Cortex in Patients with Unresponsive Wakefulness Syndrome: Preliminary Results. Neurorehabilit. Neural Repair.

[76] Xia X., Liu Y., Bai Y., Liu Z., Yang Y., Guo Y., Xu R., Gao X., Li X., He J. (2017). Long-lasting repetitive transcranial magnetic stimulation modulates electroencephalography oscillation in patients with disorders of consciousness. Neuroreport.

[77] Fan J., Zhong Y., Wang H., Aierken N., He R. (2022). Repetitive transcranial magnetic stimulation improves consciousness in some patients with disorders of consciousness. Clin. Rehabil..

[78] Manganotti P., Formaggio E., Storti S.F., Fiaschi A., Battistin L., Tonin P., Piccione F., Cavinato M. (2013). Effect of High-Frequency Repetitive Transcranial Magnetic Stimulation on Brain Excitability in Severely Brain-Injured Patients in Minimally Conscious or Vegetative State. Brain Stimul..

[79] Cincotta M., Giovannelli F., Chiaramonti R., Bianco G., Godone M., Battista D., Cardinali C., Borgheresi A., Sighinolfi A., D’Avanzo A.M. (2015). No effects of 20 Hz-rTMS of the primary motor cortex in vegetative state: A randomised, sham-controlled study. Cortex.

[80] Xu C., Zhu Z., Wu W., Zheng X., Zhong H., Huang X., Xie Q., Qian X. (2023). Effects of 10 Hz individualized repetitive transcranial magnetic stimulation on patients with disorders of consciousness: A study protocol for an exploratory double-blind crossover randomized sham-controlled trial. Trials.

[81] Xia X., Wang Y., Li C., Li X., He J., Bai Y. (2019). Transcranial magnetic stimulation-evoked connectivity reveals modulation effects of repetitive transcranial magnetic stimulation on patients with disorders of consciousness. Neuroreport.

[82] Ilmoniemi R.J., Kičić D. (2010). Methodology for Combined TMS and EEG. Brain Topogr..

[83] Gosseries O., Di H., Laureys S., Boly M. (2014). Measuring Consciousness in Severely Damaged Brains. Annu. Rev. Neurosci..

[84] Massimini M., Ferrarelli F., Huber R., Esser S.K., Singh H., Tononi G. (2005). Breakdown of Cortical Effective Connectivity During Sleep. Science.

[85] Ferrarelli F., Massimini M., Sarasso S., Casali A., Riedner B.A., Angelini G., Tononi G., Pearce R.A. (2010). Breakdown in cortical effective connectivity during midazolam-induced loss of consciousness. Proc. Natl. Acad. Sci. USA.

[86] Ragazzoni A., Pirulli C., Veniero D., Feurra M., Cincotta M., Giovannelli F., Chiaramonti R., Lino M., Rossi S., Miniussi C. (2013). Vegetative versus Minimally Conscious States: A Study Using TMS-EEG, Sensory and Event-Related Potentials. PLoS ONE.

[87] Casali A.G., Gosseries O., Rosanova M., Boly M., Sarasso S., Casali K.R., Casarotto S., Bruno M.-A., Laureys S., Tononi G. (2013). A Theoretically Based Index of Consciousness Independent of Sensory Processing and Behavior. Sci. Transl. Med..

[88] Casarotto S., Comanducci A., Rosanova M., Sarasso S., Fecchio M., Napolitani M., Pigorini A., Casali A.G., Trimarchi P.D., Boly M. (2016). Stratification of unresponsive patients by an independently validated index of brain complexity. Ann. Neurol..

[89] Bai Y., Xia X., Kang J., Yin X., Yang Y., He J., Li X. (2016). Evaluating the Effect of Repetitive Transcranial Magnetic Stimulation on Disorders of Consciousness by Using TMS-EEG. Front. Neurosci..

[90] Wang Y., Niu Z., Xia X., Bai Y., Liang Z., He J., Li X. (2022). Application of Fast Perturbational Complexity Index to the Diagnosis and Prognosis for Disorders of Consciousness. IEEE Trans. Neural Syst. Rehabil. Eng..

[91] Zhang X.-H., Han P., Zeng Y.-Y., Wang Y.-L., Lv H.-L. (2021). The Clinical Effect of Repetitive Transcranial Magnetic Stimulation on the Disturbance of Consciousness in Patients in a Vegetative State. Front. Neurosci..

[92] Pape T.L.B., Herrold A.A., Livengood S.L., Guernon A., Weaver J.A., Higgins J.P., Rosenow J.M., Walsh E., Bhaumik R., Pacheco M. (2020). A Pilot Trial Examining the Merits of Combining Amantadine and Repetitive Transcranial Magnetic Stimulation as an Intervention for Persons With Disordered Consciousness After TBI. J. Head Trauma Rehabil..

[93] Nitsche M.A., Cohen L.G., Wassermann E.M., Priori A., Lang N., Antal A., Paulus W., Hummel F., Boggio P.S., Fregni F. (2008). Transcranial direct current stimulation: State of the art 2008. Brain Stimul..

[94] Cabral M.E., Baltar A., Borba R., Galvão S., Santos L., Fregni F., Monte-Silva K. (2015). Transcranial Direct Current Stimulation: Before, during, or after Motor Training?. Neuroreport.

[95] George M.S., Ketter T.A., Post R.M. (1994). Prefrontal cortex dysfunction in clinical depression. Depression.

[96] Zhang Y., Song W. (2017). Transcranial direct current stimulation in disorders of consciousness: A review. Int. J. Neurosci..

[97] Angelakis E., Liouta E., Andreadis N., Korfias S., Ktonas P., Stranjalis G., Sakas D.E. (2013). Transcranial Direct Current Stimulation Effects in Disorders of Consciousness. Arch. Phys. Med. Rehabil..

[98] Paulus W. (2003). Chapter 26 Transcranial direct current stimulation (tDCS). Suppl. Clin. Neurophysiol..

[99] Boggio P.S., Bermpohl F., Vergara A.O., Muniz A.L., Nahas F.H., Leme P.B., Rigonatti S.P., Fregni F. (2007). Go-no-go task performance improvement after anodal transcranial DC stimulation of the left dorsolateral prefrontal cortex in major depression. J. Affect. Disord..

[100] Boggio P.S., Ferrucci R., Rigonatti S.P., Covre P., Nitsche M., Pascual-Leone A., Fregni F. (2006). Effects of transcranial direct current stimulation on working memory in patients with Parkinson’s disease. J. Neurol. Sci..

[101] Borckardt J.J., Linder K.J.B., Ricci R., Li X., Anderson B.R., Arana A.B., Nahas Z., Amassian V.M., Long J., George M.S. (2009). Focal Electrically Administered Therapy. J. ECT.

[102] Datta A., Elwassif M., Bansal V., Diaz J., Battaglia F., Bikson M. (2008). A System and Device for Focal Transcranial Direct Cur-rent Stimulation Using Concentric Ring Electrode Configurations. Brain Stimul. Basic Transl. Clin. Res. Neuromodulation.

[103] Doemkes S., Karaköse T., Antal A., Liebetanz D., Lang N., Tergau F., Cabibel V., Muthalib M., Teo W.-P., Perrey S. (2007). Shaping the Effects of Transcranial Direct Current Stimulation of the Human Motor Cortex. J. Neurophysiol..

[104] Radman T., Ramos R., Brumberg J., Bikson M. (2008). Role of cortical cell type and neuronal morphology in electric field stimulation. Brain Stimul..

[105] Accornero N., Voti P.L., La Riccia M., Gregori B. (2006). Visual evoked potentials modulation during direct current cortical polarization. Exp. Brain Res..

[106] Fregni F., Pascual-Leone A. (2007). Technology Insight: Noninvasive brain stimulation in neurology—perspectives on the therapeutic potential of rTMS and tDCS. Nat. Clin. Pract. Neurol..

[107] Sánchez-Kuhn A., Pérez-Fernández C., Cánovas R., Flores P., Sánchez-Santed F. (2017). Transcranial direct current stimulation as a motor neurorehabilitation tool: An empirical review. Biomed. Eng. Online.

[108] Hordacre B., Moezzi B., Ridding M.C. (2018). Neuroplasticity and network connectivity of the motor cortex following stroke: A transcranial direct current stimulation study. Hum. Brain Mapp..

[109] Bai Y., Xia X., Kang J., Yang Y., He J., Li X. (2017). TDCS modulates cortical excitability in patients with disorders of consciousness. NeuroImage Clin..

[110] Carrière M., Mortaheb S., Raimondo F., Annen J., Barra A., Fossati M.C.B., Chatelle C., Hermann B., Martens G., Di Perri C. (2020). Neurophysiological Correlates of a Single Session of Prefrontal tDCS in Patients with Prolonged Disorders of Consciousness: A Pilot Double-Blind Randomized Controlled Study. Brain Sci..

[111] Aloi D., della Rocchetta A.I., Ditchfield A., Coulborn S., Fernández-Espejo D. (2021). Therapeutic Use of Transcranial Direct Current Stimulation in the Rehabilitation of Prolonged Disorders of Consciousness. Front. Neurol..

[112] Schiff N.D. (2015). Cognitive Motor Dissociation Following Severe Brain Injuries. JAMA Neurol..

[113] Thibaut A., Bruno M.-A., LeDoux D., Demertzi A., Laureys S. (2014). tDCS in patients with disorders of consciousness: Sham-controlled randomized double-blind study. Neurology.

[114] Hermann B., Raimondo F., Hirsch L., Huang Y., Denis-Valente M., Pérez P., Engemann D., Faugeras F., Weiss N., Demeret S. (2020). Combined behavioral and electrophysiological evidence for a direct cortical effect of prefrontal tDCS on disorders of consciousness. Sci. Rep..

[115] Cavaliere C., Aiello M., Di Perri C., Amico E., Martial C., Thibaut A., Laureys S., Soddu A. (2016). Functional Connectivity Substrates for tDCS Response in Minimally Conscious State Patients. Front. Cell. Neurosci..

[116] Ma H., Zhao K., Jia C., You J., Zhou M., Wang T., Huang C. (2023). Effect of transcranial direct current stimulation for patients with disorders of consciousness: A systematic review and meta-analysis. Front. Neurosci..

